# Results after 10 years of colorectal cancer screenings in Spain: Hospital incidence and in-hospital mortality (2011–2016)

**DOI:** 10.1371/journal.pone.0228795

**Published:** 2020-02-10

**Authors:** Josep Darbà, Alicia Marsà

**Affiliations:** 1 Department of Economics, Universitat de Barcelona, Barcelona, Spain; 2 Department of Health Economics, BCN Health Economics & Outcomes Research S.L, Barcelona, Spain; Chang Gung Memorial Hospital at Linkou, TAIWAN

## Abstract

**Background:**

Colorectal cancer incidence in Spain increased considerably between the early nineties and 2010. To reverse this tendency, screenings were progressively implemented starting the year 2001, targeting the population aged 50 to 69 years.

**Objectives:**

This study aimed to update colorectal cancer incidence and mortality trends in Spain and provide a detailed analysis of disease management and risk factors involved in in-hospital mortality.

**Methods:**

To this aim, anonymised primary and specialised care admission records from 2011 to 2016 were extracted from a Spanish claims database representative of all Spanish regions.

**Results:**

Primary care files from 37,317 patients and specialised care files from 192,048 patients were obtained, in which males represented the 56.17% and 60.70% of patients respectively. In-hospital mortality rate was 10.07% and remained stable during the study period, similarly to colorectal cancer incidence within the hospitalised population, which was 106 per 10,000 patients. Patients deceased during the hospitalisation presented an increased presence of metastatic tumours. Mean length of hospital stay decreased significantly over the study period from 13.43 days to 11.67 days (p<0.001), similarly to patients’ 30-day readmission rate, which registered a decrease from the 15.29% to 13.58% (p<0.001). In consequence, the direct medical cost measured per patient, of €10,992, decreased over time. The implementation of colorectal cancer screening programmes caused a significant decrease in the number of new diagnoses in patients aged 75 to 79 years that may be attributable to the implementation of colorectal cancer screening programmes; however, in-hospital mortality was not reduced. Metastatic tumours and other conditions as anaemia are associated with higher in-hospital mortality rates.

## Introduction

Colorectal cancer is the third most common cancer type worldwide, with a global age-standardised incidence rate of 19.7 per 100,000 persons in 2018 [1]; in Europe, 30.0 cases per 100,000 were reached in 2018 [1].

The number of new colorectal cancer cases has increased over time in correlation with several lifestyle and environmental factors such as smoking habits, alcohol consumption, diet quality, obesity or physical activity [2]. In Spain, the age-standardised incidence rate of colon cancer increased 12.63 points between 1993 and 2010 in males, 3.51 points rectal cancer, whereas in females both rates increased 2.22 points and 1.79 points respectively [3]. In addition, malignant neoplasms of the colon and rectum were responsible for 9.2% of all cancer-related deaths in 2018, only surpassed by lung cancer [4].

Given the increasing relevance of colorectal cancer, most European countries including Spain are developing population screening programmes centred on a specific target population, in accordance with the European guidelines for quality assurance in colorectal cancer screening and diagnosis [5]. In Spain, colorectal cancer screenings were implemented the year 2001 in three regions and were progressively extended to the whole country [6]. Currently, screenings target the population aged 50 to 69 years, and reached participation rates of 45.53% in 2016 that follow an increasing tendency [7]. The positive outcome of such programmes has been measured in several occasions; colorectal malignant neoplasms diagnosed via population screening programme present more favourable histopathological features than those diagnosed based on symptoms [8]. Additionally, their positive impact has been measured as a modest decrease in colorectal cancer mortality, especially in populations with a higher risk [9, 10]. On the other hand, the implementation of such programmes has led to measurable decreases in colorectal cancer incidence in certain regions [11].

Previous evaluations of the Spanish population showed a growing incidence of colorectal cancer in the country starting the year 1975 [12]; whereas a recent study focused on colorectal cancer mortality revealed increased mortality rates among male patients [13]. These analyses of the population at risk have directed the implementation of the aforementioned screening programmes. However, a deeper understanding of the factors that influence colorectal cancer risk in Spain will be relevant for the implementation of prevention programmes, while the analysis of disease management and the aspects that determine disease prognosis can contribute to decrease mortality rates.

Thus, the objective of this study was to update disease incidence and mortality trends in Spain after ten years of screenings, and to provide a description of disease management and the factors that may play a role in colorectal cancer in-hospital mortality.

## Methods

### Study design and setting

A retrospective multicentre observational study was set to analyse primary and specialised care (hospital and other specialised care centres) records of patients admitted with colorectal cancer in Spain. Records were obtained from a claims database from the Spanish Ministry of Health that is representative of all Spanish regions. Data inclusion was set to comprise most recent available data at the moment of the analysis, from 1 Jan 2011 to 31 Dec 2016.

### Data extraction

The database, managed by the Spanish Ministry of Health, was codified at the healthcare centre level by means of the 9th revision of the International Statistical Classification of Diseases and Related Health Problems, Clinical Modification (ICD-9-CM) until 2015 and the 10th revision (ICD-10-CM) after 2016. The International Classification of Primary Care second edition (ICPC-2) was used to codify primary care records. Thus, the records of patients admitted with colorectal cancer as admission motive in specialised care centres were petitioned using the corresponding ICD-9-CM and ICD-10-CM codes, whereas to obtain primary care records data was selected via the appropriate ICPC-2 codes (Table 1).

**Table 1:** Codes used for primary care and specialised care data extraction.

Code	Definition	Coding system	Time period	Setting
D75	Malignant neoplasm of colon and rectum	ICPC-2	2011–2016	Primary care
153.0 to 153.9	Malignant neoplasm of colon	ICD-9-CM	2011–2015	Specialised care
154.0 to 154.8	Malignant neoplasm of rectum rectosigmoid junction and anus	ICD-9-CM	2011–2015	Specialised care
C18.0 to C18.9	Malignant neoplasm of colon	ICD-10-CM	2016	Specialised care
C19	Malignant neoplasm of rectosigmoid junction	ICD-10-CM	2016	Specialised care

Data extraction is conducted by the Spanish Ministry of Health that selects and provides the files of interest. Only anonymised, de-identified data is obtained, in accordance with the principles of Good Clinical Practice and the Declaration of Helsinki. This research did not involve human participants and there was no access to identifying information; in this context the Spanish legislation does not require patient consent and ethics committee approval [14].

### Study variables

The database contains raw information registered on admission detailing the patient profile and admission details. The primary care database registers patients’ sex, age, income level and employment status, centre location (Spanish region), date of admission and admission motive. The specialised care database registers patients’ sex and age, hospital location (Spanish region), date of admission, type of admission, date of discharge, type of discharge (including death), service that discharged the patient, length of stay, readmission rate, admission motive, secondary diagnoses registered during the admission, tumour morphology (codified with the International Classification of diseases for Oncology (ICD-O-3) codes), medical procedures performed and cost of the admission.

### Data analysis

In all cases, only the admissions with colorectal cancer listed as admission motive were analysed. The extraction of single-patient data for the characterisation of the patient population in both primary and specialised care was based on the first admission per patient due to colorectal cancer. Direct medical costs were calculated by using the standardised average expenses of admissions and medical procedures determined by the Spanish Ministry of Health indicated in the dataset. This calculation included all expenses related to specialised care admissions: treatment (examination, medication and surgery), nutrition, costs associated to personnel, medical equipment and resources corrected per groups of patients.

Frequencies and percentages are presented for dichotomous variables and mean and standard deviation or error were calculated for quantitative variables. Incidence rate within the hospitalised population was calculated as the proportion of patients admitted with colorectal cancer as the admission motive within the specialised care database. The characteristics of deceased patients were analysed for the admission in which death was registered. To assess the association of secondary conditions with in-hospital mortality, odd ratios (OR) with 95% confidence interval (CI) were used, with the group of patients non-deceased during the hospitalisation as the reference group. Two-tailed T-student or one-way analysis of variance were used as appropriate and two-sample Z tests were used to test for differences in sample proportions, with a p<0.05 considered statistically significant.

Data presentation is mainly descriptive. Statistical analyses were performed using Microsoft Excel© Professional Plus 2010 (Microsoft Corporation, Redmond, WA, USA) and StataSE 12 for Windows (StataCorp LP. 2011. Stata Statistical Software: Release 12. College Station, TX, USA).

## Results

The ICPC-2 code corresponding to colorectal cancer was used to extract 99,653 primary care admission records, corresponding to 37,317 single patients admitted with this cancer type between 2011 and 2016. The 56.17% of these patients were males and 43.83% females. Specialised care files were extracted via the equivalent ICD-9-CM and ICD-10-CM codes. In total, records of 230,361 admissions were obtained, corresponding to 192,048 single patients. Of these, 60.70% were males, 64.76% when only rectum cancer was considered.

The number of single patients attended per year slightly increased over the study period, with 31,531 attended in 2011 versus 33,181 attended in 2016, yet, the incidence of colorectal cancer within the hospitalised population was stable between 2011 and 2015, averaging 106.39 per 10,000 patients. The year 2016 an incidence of 88.05 per 10,000 was estimated.

**Fig 1:**
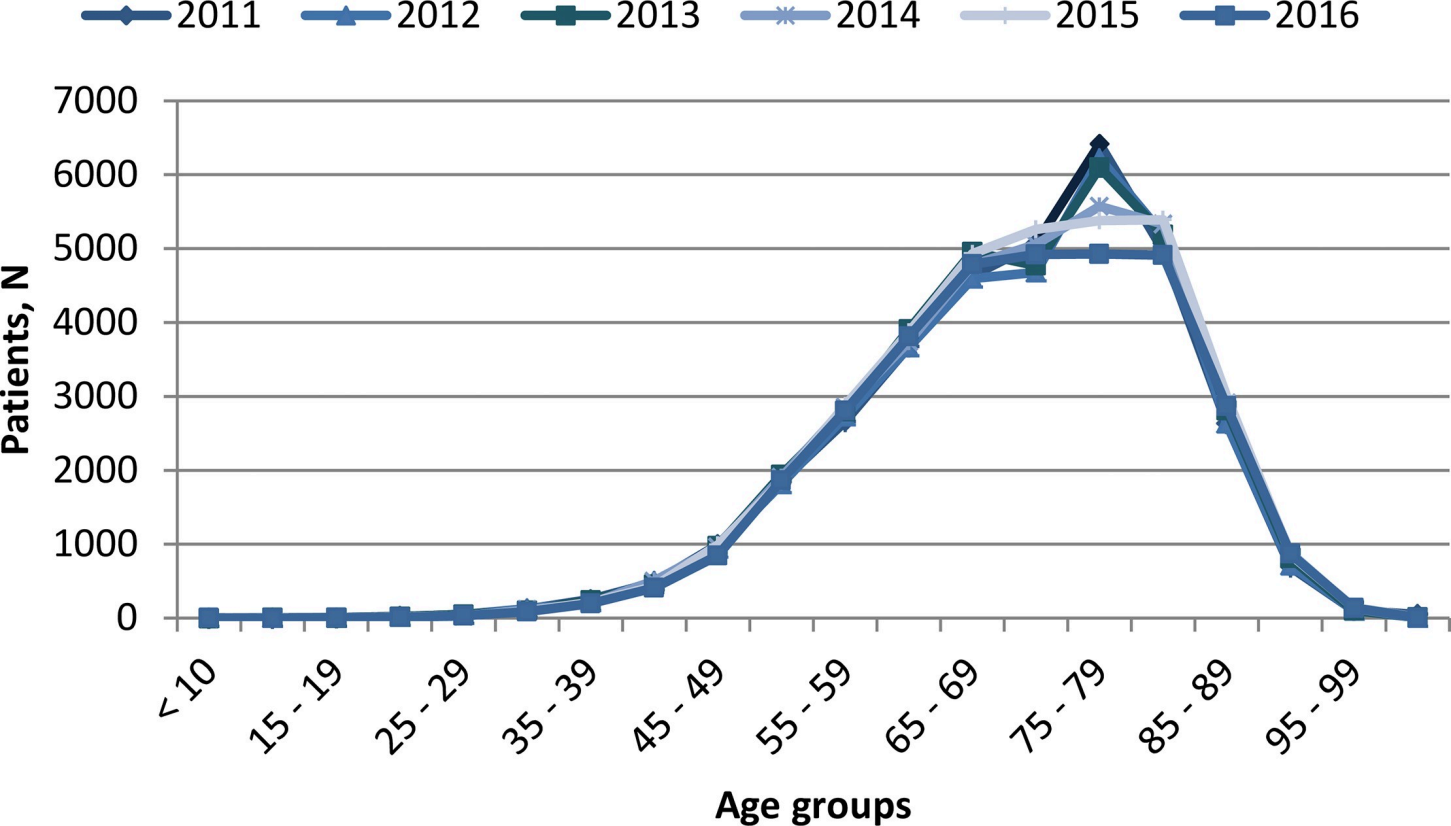
Patients’ age at first hospital admission per colorectal cancer the years 2011–2016.

Patients’ age at the first registered admission per colorectal cancer was 70.40 years (SD = 13.89) in primary care and 70.52 years (SD = 12.02) in specialised care. Overall, patients’ age distribution analysis showed a peak in the age range of 75 to 79 years, that was especially noticeable the year 2011 (Fig 1). The year 2016 the portion of patients aged 75 to 79 years was reduced 1.3 folds (p<0.001), compensated with small increases in younger patients (aged 55 to 69) and similar raises in patients aged 90 to 99.

The data registered in primary care centres included descriptive data of patients’ income level and employment status. Patients with an income under €18,000 represented 69.12% of all registries and 27.06% were between €18,000 and €99,000. Consistently with patients’ age, 67.09 of registered patients were pensioners, whereas 17.73% were active workers. Patients not active or unemployed summed the 15.19%.

Hospitalisation and specialised care records allowed the analysis per tumour type. Seventy-two per cent of cancers were malignant neoplasms of the colon and 27.88% were found in the rectum, rectosigmoid junction or anus (Table 2).

**Table 2:** Percentage and characteristics of patients diagnosed with malignant neoplasms of colon, rectum or neuroendocrine colorectal tumours.

Tumour classification	Patients, %	Males, %	Age, mean (SD)
All patients (N = 192,048)	-	60.70	70.52 (12.02)
Malignant neoplasm of colon (N = 138,514)	72.12	59.14	71.25 (11.92)
alignant neoplasm of rectum, rectosigmoid junction and anus (N = 53,534)	27.88	64.76	68.90 (12.04)
Patients with a specified neoplasm (N = 115,192)	-	60,72	71.02 (12.00)
Adenocarcinoma (N = 99,159)	86.00	61.16	70.64 (11.80)
Metastatic carcinoma (N = 9,895)	8.58	56.62	73.52 (13.20)
Carcinoma, unspecified (N = 5,743)	4.98	59.55	73.16 (12.60)
Adenoma (N = 395)	0.34	68.61	71.63 (11.09)

In addition, the majority of specified malignant neoplasms of colon and rectum were adenocarcinomas (86.00%), and 8.58% of the patients were diagnosed with a metastatic tumour during the first hospitalisation. Interestingly, an in-hospital mortality rate of 10.07% was measured during the study period, with no significant trends observed over time or in the temporal analysis stratified per patients’ age. Males and females showed similar mortality rates, 10.15% vs. 9.96%. Mortality increased with patients’ age; in patients <71 years, the mortality rate was 7.10% while in patients >71 years it was 12.64% (p<0.001). In those older than 80 years of age in-hospital mortality was 15.99% (p<0.001 vs. <80). Variation was also observed in an individual analysis per Spanish region; patients in Cantabria, Catalonia and La Rioja displayed the lowest in-hospital mortality rates (7.59%, 7.99% and 8.81% respectively), whereas the highest were measured in Ceuta and Melilla and the Canary Islands (18.17% and 17.85%) (Table 3).

**Table 3:** Analysis of patient profile and in-hospital mortality per Spanish region.

Spanish region	N	Age	% malignant neoplasms of colon	% malignant neoplasms of rectum	% in-hospital mortality
Andalusia	29,273	70.72	66.65	33.35	12.49
Aragon	6,560	70.53	66.72	33.28	12.20
Principality of Asturias	6,142	70.77	66.93	33.07	12.55
Balearic Islands	3,058	70.18	65.34	34.66	10.53
Canary Islands	6,567	70.56	66.59	33.41	17.85
Cantabria	2,627	70.60	66.88	33.12	7.59
Castille and León	15,446	70.62	66.35	33.65	12.72
Castilla—La Mancha	8,220	70.46	66.80	33.20	12.38
Catalonia	34,822	70.60	67.16	32.84	7.99
Valencian Community	25,008	70.39	66.64	33.36	10.86
Extremadura	4,872	70.50	66.36	33.64	12.49
Galicia	16,162	70.68	67.08	32.92	11.50
Community of Madrid	23,930	70.67	66.79	33.21	10.28
Region of Murcia	5,124	70.61	66.41	33.59	10.20
Chartered Community of Navarre	3,421	70.41	66.09	33.91	12.52
Basque Autonomous Communty	11,465	70.47	66.25	33.75	12.42
La Rioja	1,462	70.59	65.25	34.75	8.81
Ceuta and Melilla	373	71.99	67.57	32.43	18.17

Independently, secondary diagnoses registered upon admission were used to assess the effect of comorbid conditions in colorectal cancer mortality. With all admissions considered, metastatic malignant neoplasms, mainly of the liver, were found in virtually all patients deceased during the hospitalisation (Table 4). The portion of metastatic neoplasms of the retroperitoneum and the lung were also increased in this group, found in 16.81% and 20.55% of all deceased patients respectively.

**Table 4:** Secondary diagnoses in patients with colorectal cancer and in deceased patients.

Secondary diagnoses	All patients, %	Deceased patients, %	Odds ratio, 95% CI
Metastatic malignant neoplasm	57.38	100.00	2.58 (2.53–2.63)
Liver	15.04	37.37	2.92 (2.84–3.00)
lymph nodes	10.43	8.85	0.82 (0.78–0.86)
Lung	6.38	20.55	4.20 (4.04–4.36)
retroperitoneum	6.00	16.81	3.44 (3.31–3.58)
Essential hypertension	33.29	28.32	0.82 (0.80–0.84)
Disorders of lipoid metabolism	18.74	12.82	0.65 (0.62–0.67)
Diabetes mellitus	16.79	15.80	0.92 (0.88–0.95)
Intestinal obstruction without mention of hernia	13.43	20.29	1.57 (1.52–1.63)
Anaemia	10.86	12.20	1.12 (1.07–1.16)
Cardiac dysrhythmias	8.72	13.60	1.63 (1.56–1.70)
Tobacco use disorder	7.47	6.60	0.86 (0.81–0.90)
Overweight and obesity	7.39	4.03	0.51 (0.48–0.54)

The overall mean length of hospital stay (LOHS) decreased significantly over time from 13.43 days (SE = 0.07) in 2011 to 11.67 days (SE = 0.08) in 2016 (p<0.001), as it happened with the readmission rate, which decreased from 15.29% to 13.58% (p<0.001). Altogether, 41.21% of all the admissions included in the study were registered as non-scheduled or urgent, and 99.63% required inpatient care.

General and digestive surgery and other surgery services were responsible for patients’ discharge in 70.50% of admissions, and patients’ destination after discharge was principally their home. Patients deceased during hospitalisation were treated by internal medicine and oncology services, in addition to palliative care units.

The hospital and specialised care database permits the calculation of hospitalisation costs, which considers the estimated cost per all medical procedures performed, equipment and other factors such as patients’ condition. In order to provide a more detailed evaluation of medical costs, the most relevant registered medical procedures were investigated (Table 5). Various surgical resection and excision procedures were used, while the infusion of chemotherapy was registered in only 11.53% of all admissions.

**Table 5:** Principal medical procedures performed in the course of the hospital admission.

Medical procedures	% of admissions
Imaging	74.99
computerized axial tomography of abdomen	30.55
computerized axial tomography of thorax	11.64
routine chest x-ray	8.24
ultrasound of abdomen and retroperitoneum	7.67
Biopsies	17.79
closed [endoscopic] biopsy of large intestine	13.72
Chemotherapy	11.53
Surgery	-
open and other right hemicolectomy	12.92
other anterior resection of rectum	10.37
other small-to-large intestinal anastomosis	9.06
open and other sigmoidectomy	8.00
Other	-
transfusion of packed cells	10.89
injection of antibiotic	10.03

**Fig 2:**
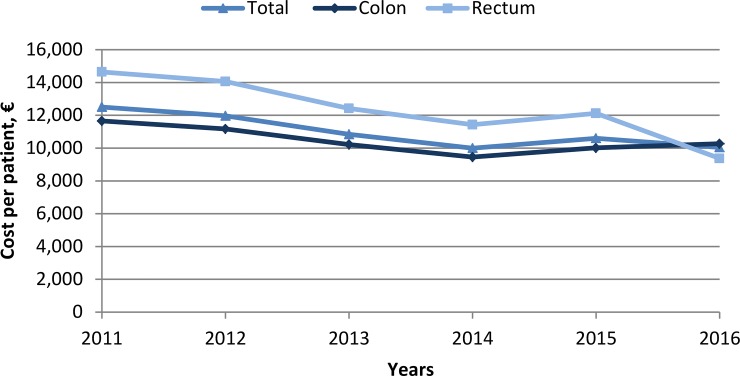
Mean direct medical cost of specialised care per patient measured over time.

Finally, taking the aforementioned factors into account, the direct medical cost measured per admission averaged €10,992, with a decreasing tendency observed over time (p<0.001) (Fig 2). The mean direct medical cost per patient in those with a tumour of the colon was €10,464, whereas the mean cost per patient with a rectal tumour was €12,339. Clear tendencies were not observed when analysing costs stratified per patients’ age. A decrease in the cost per patient was observed the year 2016.

## Discussion

The analysis of primary and specialised care files of patients diagnosed with colorectal cancer in Spain revealed an increased number of new cases among males, mostly neoplasms of the colon, which is comparable to data obtained in other countries [15]. In Spain, screening programmes have been progressively implemented starting the year 2001 with only three regions and expanding, including individuals aged 50 to 69 years [5, 12]. The reduction observed in this study in the number of patients aged 75 to 79 years between the years 2011 and 2016 could be explained by the increased early detection of these tumours as a consequence of the implementation of such screening programmes. Incidence among the hospitalised population was relatively elevated, with 106.39 per 10,000 patients between 2011 and 2015, and remained stable during the study period. Incidence decreased in 2016, presumably due to the introduction of ICD-10 coding, indicating the need to interpret this data cautiously.

In addition, it is estimated that approximately 20% of the patients with colorectal cancer present with metastasis at the time of diagnosis [16], a rate that was smaller in the present study, with only the 8.58%, although the use of other ICD codes to codify these cases cannot be discarded. The diagnosis of metastatic tumours among colorectal cancer patients during the first admission remarks the importance of early detection, achieved via screening programmes that should be promoted to achieve higher participation rates. Its utility has been proven worldwide and in the country [17]; indeed, one study analysing key performance indicators of the first screening years in Spain indicated that the majority of cancers were detected at an early stage [18].

In addition, in-hospital mortality was seemingly elevated, with no significant trends observed over time. Previous inpatient mortality estimations in Spain measured comparable rates, between 10 and 11%, yet, measurements in other countries situated those rates under the 3% when only taking into account post-surgery mortality [13, 19, 20]. Data obtained herein suggests that in-hospital mortality is not directly related to surgical procedures; instead, most deceased patients were registered in oncology services or palliative care units. On the other hand, the presence of certain comorbid conditions could play a role in in-hospital mortality. Metastatic tumours were present in virtually all deceased patients; likewise, anaemia and intestinal obstruction were increased in these patients. Anaemia is frequently observed in colorectal cancer patients and preoperative anaemia has been associated with poor survival [21]. Herein it appears to have an effect in disease course; nevertheless, its relation with preoperative indicators has not been investigated. The influence of diet quality, obesity and physical activity in tumour progression, is not apparent in this study, which could be a consequence of the weight loss observed in the last stages of the disease [2, 22]. It must be taken into account that secondary conditions observed in deceased patients are registered during the hospitalisation process in which the patient dies. This could explain the negative relation found between certain conditions as essential hypertension, disorders of lipoid metabolism, overweight and obesity and in-hospital mortality. As for diabetes, the modest protective effect found in this study is in contradiction with previous meta-analyses, which suggested a negative effect of this condition on colorectal cancer survival, and must be interpreted with caution [23].

Regarding disease management, data in Spain showed a tendency to decrease hospitalisation times, which has been followed by a reduction in readmission rates. Such tendencies could reflect the application of more efficient treatment and recovery protocols, and therapies that take into account tumour histopathological characteristics [24, 25]; in addition, the introduction of screening programmes may lead to earlier cancer detection, requiring less complex treatments and with fewer complications [18]. Previous evaluations determined that patients above 80 years of age receive similar treatment than younger patients, yet, they required the longest hospitalisation times, which is presumably associated to the presence of comorbidities and other age-related complications [26].

Finally, an assessment of direct medical costs was performed. Mean cost per patient was €10,992 during the study period, with a decreasing tendency observed over time. Yet, the marked reduction measured the year 2016 could be related to the shift to ICD-10 coding. Further research will be required to determine whether this decrease can be associated to the introduction of screening programmes and the earlier detection of this cancer type [27]. As a reference, the public healthcare cost per person in Spain was approximately €1,600 the year 2017, with 63.2% of total costs dedicated to specialised care [28]. The costs measured herein are likely to be derived from surgical procedures, which were estimated to account for up to 59.2% of all costs of colorectal cancer patients in one Spanish hospital [29].

In the same way, differences in costs between cancer types and stages, patients age and comorbidity status have been documented in several occasions and should be taken into account in future studies for the optimisation of healthcare protocols [30, 31].

A number of limitations may have influence in the results obtained. ICD-9-CM and ICD-10-CM codes did not allow differentiation between the distinct stages of colorectal cancer, preventing further analyses; in addition, the introduction of ICD-10 codification the year 2016 represented a source of error due to the gradual implementation of this codification.

## Conclusions

The implementation of colorectal cancer screening programmes in Spain presumably provoked the decrease in the number of new diagnoses in patients aged 75 to 79 years; however, in-hospital mortality remained stable. Such programmes should be promoted to achieve higher participation rates in an effort to reduce incidence and the number of patients presenting metastasis at first admission. The roles of anaemia and overweight in colorectal cancer mortality should be further investigated to determine their value in improving patients’ prognosis.
